# Most Pathways Can Be Related to the Pathogenesis of Alzheimer’s Disease

**DOI:** 10.3389/fnagi.2022.846902

**Published:** 2022-06-24

**Authors:** Sarah L. Morgan, Pourya Naderi, Katjuša Koler, Yered Pita-Juarez, Dmitry Prokopenko, Ioannis S. Vlachos, Rudolph E. Tanzi, Lars Bertram, Winston A. Hide

**Affiliations:** ^1^Harvard Medical School Initiative for RNA Medicine, Department of Pathology, Beth Israel Deaconess Medical Center, Boston, MA, United States; ^2^Harvard Medical School, Boston, MA, United States; ^3^Blizard Institute, Department of Neuroscience, Surgery and Trauma, Queen Mary University of London, London, United Kingdom; ^4^Department of Computer Science, The University of Sheffield, Sheffield, United Kingdom; ^5^Broad Institute of MIT and Harvard, Cambridge, MA, United States; ^6^Genetics and Aging Research Unit, The Henry and Allison McCance Center for Brain Health, Department of Neurology, Massachusetts General Hospital, Boston, MA, United States; ^7^Lübeck Interdisciplinary Platform for Genome Analytics, Institutes of Neurogenetics and Cardiogenetics, University of Lübeck, Lübeck, Germany; ^8^Center for Lifespan Changes in Brain and Cognition, Department of Psychology, University of Oslo, Oslo, Norway

**Keywords:** Alzheimer’s disease, pathway, text-mining, disease mechanism, dementia

## Abstract

Alzheimer’s disease (AD) is a complex neurodegenerative disorder. The relative contribution of the numerous underlying functional mechanisms is poorly understood. To comprehensively understand the context and distribution of pathways that contribute to AD, we performed text-mining to generate an exhaustive, systematic assessment of the breadth and diversity of biological pathways within a corpus of 206,324 dementia publication abstracts. A total of 91% (325/335) of Kyoto Encyclopedia of Genes and Genomes (KEGG) pathways have publications containing an association *via* at least 5 studies, while 63% of pathway terms have at least 50 studies providing a clear association with AD. Despite major technological advances, the same set of top-ranked pathways have been consistently related to AD for 30 years, including *AD*, *immune system*, *metabolic pathways*, *cholinergic synapse*, *long-term depression*, *proteasome*, *diabetes*, *cancer*, and *chemokine signaling*. AD pathways studied appear biased: animal model and human subject studies prioritize different AD pathways. Surprisingly, human genetic discoveries and drug targeting are not enriched in the most frequently studied pathways. Our findings suggest that not only is this disorder incredibly complex, but that its functional reach is also nearly global. As a consequence of our study, research results can now be assessed in the context of the wider AD literature, supporting the design of drug therapies that target a broader range of mechanisms. The results of this study can be explored at www.adpathways.org.

## Introduction

Alzheimer’s disease (AD) is a genetically complex, heterogeneous neurodegenerative disorder that has a heavy economic and emotional burden on the society. As the most common form of dementia, it is predicted to be the cause of 43% of all older adult deaths in the United States by 2050 (Weuve et al., 2014). Our pathophysiological understanding of AD has been evolving rapidly in recent years, driven in part by the clinical trial failures of drugs designed to block AD-related pathology. Although many reasons for these failures have been proposed, it is evident that we simply do not understand the disease well enough (Lao et al., 2019). Functional studies, performed in animal or human-derived models, yield potential disease mechanisms that offer actionable insights into therapeutic intervention. These studies also aim to improve our understanding of AD by analyzing the functional pathways that are altered as the disease takes its course. Yet, most studies which yield a potential role for a pathway rarely assess the pathway’s relative contribution to AD. The same is true for review articles, often focusing on only a handful of pathways (Khalil et al., 2016; Butterfield and Boyd-Kimball, 2018; Hardy and Escott-Price, 2019). The most common of these include *amyloid processing* pathways, *immune system*, and *oxidative stress* related pathways. More comprehensive reviews can document 10–50 AD-associated pathways (Calabrò et al., 2021; Gadhave et al., 2021) but do not attempt to assess the total number of pathways associated with the disease.

**FIGURE 1:**
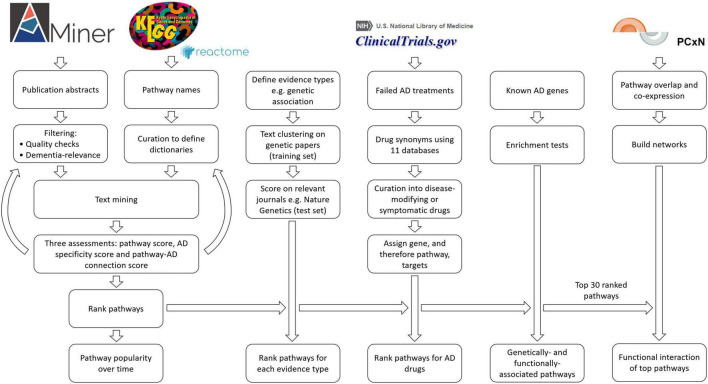
Flowchart outlining the study methods. For the filtering of publications, we kept only studies with an abstract and with the terms *Alzheimer* or *dementia*. We removed duplicates, abstracts with negative findings, abstracts with dementia as an exclusion criterion, abstracts that were too long or too short, and finally, all keywords if there were more than 30 (Supplementary Methods).

We set out to determine the relative contribution of dementia-related pathways to AD by text-mining the biomedical literature for a joint occurrence of *dementia* and a *pathway*. We chose text-mining so that we could automate the assessment of the entire AD literature (206,324 references) using the abstract of a publication as a concise summary of the main hypothesis and findings of a study. As AD is the most common form of dementia, we examined the entire dementia literature. To directly link the published evidence to AD, we concurrently scored these studies on how closely they were linked to AD, ensuring that negative results were excluded from the analysis. We scrutinized abstracts obtained using AMiner (Wan et al., 2019) to quantify the relative association of 341 biological pathways with AD (Figure 1). AMiner is an easy-to-download, open knowledge graph containing scientific publication records. The Kyoto Encyclopedia of Genes and Genomes (KEGG) was chosen for the “core” list of pathways to be examined (Kanehisa et al., 2017) along with three immune pathways from Reactome (Jassal et al., 2020). KEGG is one of the most widely used databases for enrichment studies and also one of the oldest, making it ideal for time-dependent analysis. Alternatively, structured data systems such as the Gene Ontology now have thousands of pathway terms, which unfortunately increase the complexity of text-mining. Therefore, we chose KEGG’s 335 pathways as a simplified, but manageable source for a broad range of extensively used pathway terms.

One goal of this project was to determine the evidence predicted to be driving each pathway’s association with AD. By development and benchmarking of our dictionaries on topic-specific journals, we characterized biases in the source of pathways identified by genetic, human, or animal studies. Seeking to assess the literature further, we addressed the questions of how the focus of AD research has changed in recent years, what pathways are targeted by AD drugs, what are the relationships between AD-related pathways in terms of their genes and co-expression, and to what extent pathways are enriched with AD-associated genes. This systematic assessment of the published literature reveals that almost all (91%) of the currently known KEGG biological pathways were associated with AD in at least five publications; this number dropped to 88%, 63%, and 49% when restricting evidence to 10, 50, or 100 publications, respectively.

## Materials and Methods

### Data Collection and Processing

In order to capture and assess the literature, we used the AMiner dataset as our key resource (Wan et al., 2019). The AMiner tool contains 172,209,563 publications in the period from 1740 to 2019. Each entry includes a title, abstract, year, keywords, journal, author list, DOI, and other relevant identification attributes. To improve the accuracy of our results, we performed pre-processing using a series of filtering steps (Figure 1 and Supplementary Methods).

### Pathway Dictionaries

To obtain the names of a range of biological functions to be searched, we used two pathway databases, manually curated into dictionaries (Supplementary Methods). We selected human pathways from the third level of the KEGG database (*n* = 335), as the lowest level contained greater than 40,000 pathways, many of which have overlapping terms with the 335 pathways they stem from. Enrichment tests often use this third level as well. We additionally added the pathways, namely, *immune system*, *cancer*, and *diabetes*, and three pathways from Reactome, namely, *adaptive immune system*, *innate immune system*, and *cytokine signaling in immune system*. These pathways were chosen, as KEGG is lacking in immune pathways, which we believe would score high in the AD literature. *Cancer* and *diabetes* appear in KEGG only as specific types of each disease, whereas we also wanted to know how they performed as a whole.

We developed three scoring methods to evaluate our data: (1) a pathway score, (2) an AD specificity percentage (described in the “Temporal trends in AD research” section), and (3) a score for assessing a pathway’s association to dementia based on a small sample of studies (AD-pathway score). To assess the reliability of the pathway dictionary, a minimum of 10 random abstracts were manually assessed and scored for how often the pathway of interest was the focus of the abstract (pathway score; Supplementary Methods).

Next, to assess the strength of the pathway’s association to AD or dementia, a new selection of abstracts was scrutinized to assess each pathway’s association to the disease (AD-pathway score). Per pathway, we scored between 2 and 20 studies on whether the association with AD was substantive (refer to Supplementary Table 1 for full scoring criteria). We did not require these pathway-name mentions to also reference the KEGG database. This was particularly pertinent for disease pathways wherein most studies describe the actual disorder rather than the KEGG disease pathway. Some pathways required a number of iterations of the initial dictionaries to achieve acceptable scores. Depending on which terms were found in the study, the pathway could have a full association (enough relevant words were recorded) or a partial association. We ranked all the pathways against each other based on the whole dementia literature and then individually for every year since 1990. Publications before 1990 were too sparse to be included. For the yearly ranks, we performed a hierarchical cluster analysis to group similar pathways for a number of mentions historically.

### Evidence Dictionaries

To determine the types of studies driving or providing biases in pathway associations, we created a dictionary of evidence types of 29–62 terms (Supplementary Methods). For example, a genetic evidence-based connection would contain words such as *gene, mutation*, or *variant*, while an evidence-based connection of AD model would more likely use terms such as *rat, mice*, *or transgenic*. Studies on human cells were included in the model category. The categories we examined included animal, genetic, human, *in silico*, *in vitro*, *in vivo*, model, and reviews. Review studies were excluded from the other groups, as they do not represent original research. The dictionaries were curated using text clustering on a selection of relevant AD studies (Supplementary Methods). These were then analyzed for their accuracy in assigning publications using specific journals relevant to each category, for example, the journal “*in vitro* cellular” was used to assess the *in vitro* dictionary. A study could be assigned to either of the competing evidence types or labeled as *both* or *neither*.

### Dementia Stratification

To assess the specificity of each pathway to AD, we calculated the percentage of each pathway’s studies that contain AD-specific terminology. Each study could be assigned to one of four main classes based on our dementia dictionaries (using 13–27 terms; Supplementary Methods). The first class includes highly specific terms such as *Alzheimer* or *APP*, the second class includes dementia terms such as *memory* or *aging*, the third class includes words describing related dementias such as *frontotemporal* or *vascular*, and finally the fourth one includes unrelated dementias including *viral-induced* dementia and *Creutzfeldt*. The relative proportions of these words per study were used to identify pathway associations driven by AD, related, or unrelated dementia. The suggestive terms were used to differentiate between the unrelated dementias and the other two categories: AD or related dementia. Abstracts with an uncertainty between being classified as either AD or related disease were labeled as *dementia*, as were studies with very few keywords besides *dementia* (refer to Supplemental Methods for a full description).

### Effect of Journal Impact Factor

To understand whether inclusion of all studies regardless of quality affected our pathway rankings, we used the crude measurement of journal impact factor to restrict our analysis to studies from high impact sources. We selected journals with an impact factor of greater than 5 and greater than 10, and then repeated the pathway association analysis.

### Drug Analysis

To investigate the pathways that AD drugs previously or currently target, we assessed the gene targets of proposed disease-modifying and symptomatic treatments. We downloaded all AD-associated drug trials from clinicaltrials.gov (27 September 2019). We extracted drug interventions, excluding biomarkers, leaving a total of 1,021 clinical trials. To collect every treatment’s synonyms, we constructed a drug-synonym database from a number of different drug databases, namely, ChEMBL (Gaulton et al., 2017), CMap (Lamb et al., 2006), CTD (Davis et al., 2019), DrugBank (Wishart et al., 2018), EMA (European Medicines Agency, 2019), L1000CDS2 (Duan et al., 2016), LINCS (Subramanian et al., 2017), PharmGKB (Barbarino et al., 2018), RepoDB (Brown and Patel, 2017), and Wikipedia (Wikipedia contributors, 2018). We removed non-specific drug names, for example, *calcium channel blocker*. We curated each drug as either a symptomatic indication or as disease-modifying, i.e., it was investigated with the aim of delaying the onset or slowing the progression of the disease. As clinical trials often lack this information, we used the literature and Alzforum to inform us of the aim of a drug (Supplementary Methods; Alzforum, 2021). Drugs that could not be adequately curated into either category were excluded. We next searched for the curated drugs in the Open Targets resource (Carvalho-Silva et al., 2019) and the Therapeutic Target Database (Wang et al., 2020) to assign gene targets to each drug. This enabled us to examine which pathways were the most targeted by proposed drug interventions.

### Statistical Analysis

To compare the effect of impact factor on pathway rankings, we used a Spearman’s rank-order correlation coefficient to compare the ranks from the three groups named in the “The effect of journal impact factor on pathway ranks” section.

Annotated pathways can share genes or proteins with each other and thus generate bias in the interpretation of results from (gene) member enrichment tests in high-throughput studies. To address overlap bias of analyses, we determined the degree to which AD-associated pathways overlap *via* shared genes. We used a standard enrichment analysis pipeline, using Fisher’s exact test, between all possible pairs of KEGG-only pathways with 10 or more genes to determine overlap statistics. We adjusted enrichment *p*-values for multiple hypothesis testing using false discovery rate (FDR) (Benjamini and Hochberg, 1995). We extracted an overlap network of the top 30 ranked KEGG pathways according to the overall ranking from text-mining of AD literature. In this network, nodes represent pathways and links denote whether a pair of nodes have an adjusted *p*-value of <0.05. The overlap network was processed and visualized using the *R-igraph* package (Csardi and Nepusz, 2006). The edge thickness represents the negative logarithm of the adjusted *p*-value of enrichment. The graph was visualized using a forced layout algorithm. The node colors were annotated to represent AD word score, which denotes the average number of AD-specific terms per study associated with each pathway.

We analyzed the association of pathways with known AD genes derived from two predetermined independent lists. First, we listed genes as determined by the Open Targets system (downloaded on 2 December 2020) for having an overall association score of >0.1 *via* the use of genetic associations, known drugs already used to treat the disease, and known affected pathways and significant changes in RNA expression in AD (Koscielny et al., 2017). We referred to these as AD-associated genes (*n* = 306, Supplementary Table 2). Second, we utilized an expert-curated list of 33 AD genes from familial or genome-wide association studies (GWAS; Supplementary Table 3). We refer to these as genetically defined AD genes. We determined the association of KEGG pathways and the gene lists using enrichment analysis.

### Pathway Co-expression

To determine co-expression patterns among AD-associated pathways, we used the Pathway Co-expression Network (PCxN) customized to KEGG-only pathways (Pita-Juárez et al., 2018). PCxN generates a canonical co-expression network among a given set of pathways using 3,207 gene expression profiles from 72 tissues. In PCxN, each edge represents a partial correlation between gene expression summary statistics of two pathways. Each summary statistic in PCxN is a proxy value of a pathway’s expression calculated from its member genes. The partial correlation between the two pathways is conditioned on, and corrected for, the overlap between them. We extracted the co-expression network of the top 30 literature-associated KEGG pathways. The network was processed and visualized using the *R-igraph* package (Csardi and Nepusz, 2006). Only those PCxN-edges with statistically significant correlation (FDR < 0.05) were included in the network construction. The edge thickness is proportional to the absolute value of correlation coefficient between two nodes. The correlation network was mapped to the layout from the network of the pathway overlap analysis for comparability. Given the slight differences in the background of the text-mining pathways and those annotated in PCxN, only pathways present in both datasets were included for visualization.

## Results

### Pathway Associations

We found that 325 of 335 KEGG pathways and all 6 additional pathways (*immune system*, *cancer*, *diabetes*, *adaptive immune system*, *innate immune system*, and *cytokine signaling in immune system*) have publications containing an association with AD. Of the unrelated KEGG pathways, two could be connected to dementia in general. Pathways with the strongest literature association to dementia were *Alzheimer’s disease*, *Parkinson’s disease*, *immune system* (added to our KEGG list), *metabolic pathways*, *long-term depression, cholinergic synapse*, *diabetes* (also added), and *apoptosis*. If we restrict this to AD-specific studies only, we get similar, most frequent literature association pathways, namely, *Alzheimer’s disease*, *immune system*, *metabolic pathways*, *cholinergic synapse*, *long-term depression*, *apoptosis*, *proteasome*, *diabetes*, *cancer*, and *chemokine signaling pathway* (Supplementary Table 4). The 7 pathways not related to AD or dementia were *amino sugar and nucleotide sugar metabolism*, *amoebiasis*, *antifolate resistance*, *collecting duct acid secretion*, *maturity onset diabetes of the young*, *proximal tubule bicarbonate reclamation*, and *yersinia infection*.

### Dementia Breakdown

**FIGURE 2:**
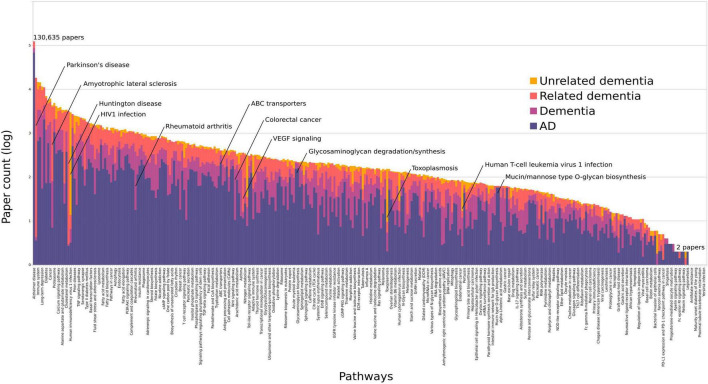
All pathways ordered by the log of their total paper counts associating them with AD or dementia. We have colored each bar to represent the percentage of papers containing an adequate number of AD-relevant words (purple), dementia terms (pink), dementia caused by related disorders (orange), and unrelated dementias (yellow). Pathways with only 1 study do not display on this scale. The average percentage of AD-specific studies per pathway was 64% excluding those with less than 50 studies. The highest proportions were found in *ABC transporters* (88%), *Alzheimer’s disease* (95%), the *glycosaminoglycan pathways* (93–94%), and the *O-glycan* pathways (98%). These data are summarized in Supplementary Table 4.

We investigated the relative proportions of pathway-assigned studies to four dementia categories to assess the specificity to AD (Figure 2). These were AD-specific, dementia, related dementia, and unrelated dementia. The Spearman’s correlation coefficient was 0.98 between the pathway ranks from all dementia studies and AD-specific studies, with only two pathways in the top 100 significantly changing their rank: *HIV1 infection* (rank change 19 to 66) and *Huntington’s disease* (rank change 18 to 74). *Infection pathways* are associated with a higher number of studies from *unrelated dementia*, due to the AIDS dementia complex, which is not directly related to AD. The Reactome *adaptive immune system* pathway had the next highest percentage of *unrelated dementia* assignments at 20%; however, this pathway was still highly associated with AD. Parkinson’s disease (PD) and amyotrophic lateral sclerosis (ALS) were both part of the *related dementia* dictionaries, given their overlap in genetics and protein misfolding with AD, hence scoring high under this category (Figure 2). Neither changed significantly in their overall rank compared with their AD-specific rank (Supplementary Table 4).

### Evidence Driving Alzheimer’s Disease Association

One of the goals of this study was to assess how comparable the prioritized pathways are from different types of studies. We examined all publications in the context of genetic vs. model; human vs. animal, and *in vivo* vs. *in vitro*. To assess the accuracy of the dictionaries, we used journals specific to each category as true positive and true negative studies. For example, studies from the *Journal of Human Genetics* should be categorized as *genetic* and not *model*, and *vice versa* for the journal Laboratory Animal. We applied the evidence dictionaries to abstracts collected from journals specific to each category (Supplementary Figure 1; Supplementary Methods).

**FIGURE 3:**
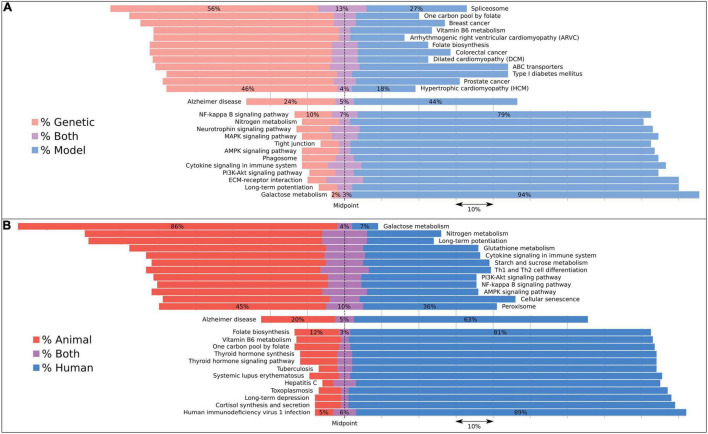
Pathways associated with AD subcategorized by evidence type using dictionaries to reveal biases in their associations. Bars are aligned along the midpoint of the neutral grouping, so that a direct comparison can be made of the two competing groups. The missing percentage from this chart is for studies assigned to neither evidence category, but this value can be inferred by subtracting the total bar quantity from 100, or found in Supplementary Table 5. We compared **(A)** genetic with model studies and **(B)** animal with human studies. We included the 12 most skewed pathways for each of these four evidence types as well as the Alzheimer’s disease pathway, which represents the AD expected rate for each category. Both human and animal studies were included under the *model* category. Pathways with less than 100 studies associating them to dementia were excluded.

Implementing these dictionary comparisons for AD-associated pathways revealed biases in a select number of pathways (Supplementary Table 5) depending upon the underlying basis for the discovery of an AD association: studies describing genetic associations include several diseases such as cardiomyopathy and some cancers (Figure 3), while the most frequent, genetically driven pathway appears to be the *spliceosome* pathway. In contrast, animal and model studies have revealed *galactose metabolism* as the most frequently associated pathway.

### Temporal Trends in Alzheimer’s Disease Research

**FIGURE 4:**
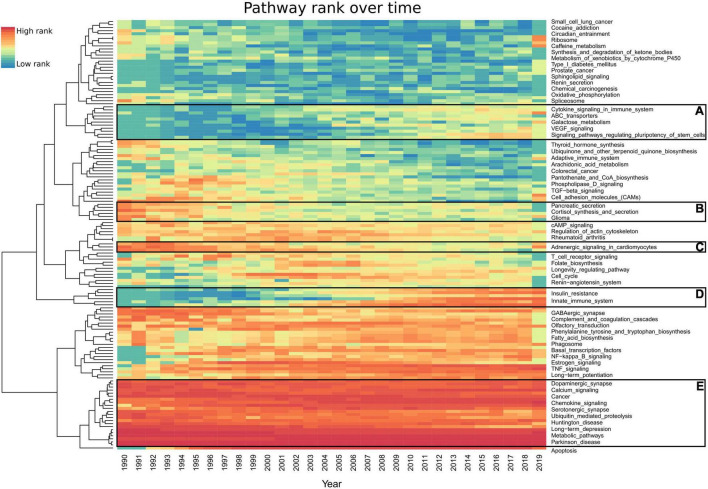
Heatmap representing each pathway’s prevalence over the last 30 years. Each year’s highest ranked pathways are denoted in red, while the lowest rank is denoted in blue. For an improved visualization, we excluded 142 pathways having less than 20 studies per year. AMiner was updated in 2019 so it only captures part of that year. **(A,D)** Pathways which gained interest. **(B,C)** Pathways which have lost interest since the 1990s. **(E)** Pathways which have been consistent in their high scores every year since 1990. Full results can be found in Supplementary Table 6.

To explore trends in dementia research, we examined the annual proportion of each pathway’s mentions (Figure 4). The most frequently ranked pathways over the last 30 years have not significantly changed (Figure 4E). The pathways with the largest increase in yearly rank since the early 1990s include *type II diabetes mellitus*, *autophagy*, *PI3K-Akt signaling*, *NF-kappa B signaling*, *mTOR signaling*, *signaling pathways regulating pluripotency of stem cells*, *innate immune system* (Reactome), *insulin resistance*, *PPAR signaling*, and *apoptosis* (Figure 4D). Pathways such as *pancreatic secretion* have seen a decrease in their rank (Figures 4B,C), while *VEGF signaling* has increased (Figure 4A). A total of 142 pathways had less than 20 studies per year (Supplementary Table 5).

### The Effect of Journal Impact Factor on Pathway Ranks

**FIGURE 5:**
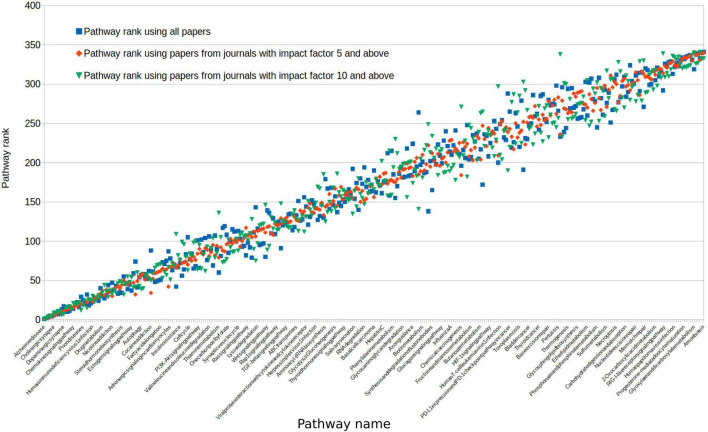
We reassessed all pathway ranks after selecting journal sources with a differing impact. Impact of greater than 5 (red) or greater than 10 (green) comparing this to all studies from any journal (blue). For clarity, pathways on this figure were ordered by the average rank across these three groups. Because of this, the variability in impact >5 (red) falsely appears to be less, as it is a halfway point between the other two scores.

To assess the impact of paper quality on pathway rankings, we repeated our analysis with impact factor restrictions. Studies sourced from high impact journals of either greater than 5 or greater than 10 did not significantly alter the rank order of pathways (Spearman rank-order correlation: 0.97 and 0.95, respectively; Figure 5). Using only studies from journals with an impact of greater than 10, 93% of pathways were still associated with dementia in at least 1 study.

### Pathway Targets From Clinical Trials

We evaluated proposed AD-modifying drugs for the pathways they commonly target to reveal mostly *disease*, *immune*, and *infection* pathways (Supplementary Table 7). Conversely, symptomatic treatments frequently target *signaling* and *synapse* pathways. The most targeted pathways by 69 disease-modifying treatments are *pathways in cancer*, *cytokine signaling in immune system*, *innate immune system*, *Alzheimer’s disease*, *serotonergic synapse*, *human papillomavirus infection*, *neuroactive ligand-receptor interaction*, *human cytomegalovirus infection*, and *metabolic pathways*. Two of these pathways are additionally the most targeted by symptomatic indications, namely, *serotonergic synapse* and *neuroactive ligand-receptor interaction* (Supplementary Table 7). Other targets for supportive therapy include *calcium signaling pathway*, *cAMP signaling pathway*, *nicotine addiction*, *dopAMinergic synapse*, *alcoholism*, *cocaine addiction*, and *cholinergic synapse*.

### Pathway Interaction and Alzheimer’s Disease-Associated Gene Enrichment

**FIGURE 6:**
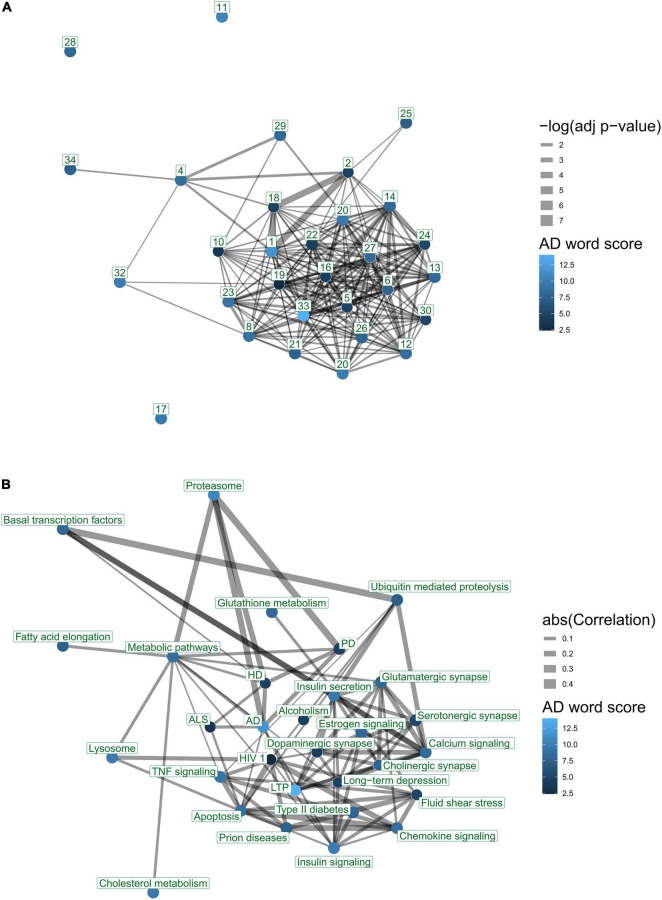
Interaction networks of top 30 KEGG pathways according to text-mining. Nodes represent pathways. Node color indicates *AD word score* denoting average number of AD-related terms per paper, for each pathway. Edges reflect a significant association between two pathways. Edge thicknesses reflect the strength of association. **(A)** Gene overlap between pathways. Edges represent significant enrichment between pairs of pathways (FDR < 0.05). Node number indicates ranking in the literature. Edge thicknesses reflect significance (–log *p*-value) of enrichment. **(B)** Co-expression of pathways. Edges represent canonical correlations as defined by a pathway correlation analysis (PCxN) (Pita-Juárez et al., 2018). Edge thickness values represent the absolute value of correlation between the two pathways. The figure was generated using the igraph package. The nodes were placed in the two-dimensional coordinates using a forced layout algorithm for the gene overlap network. The co-expression network was aligned to the same layout as the gene overlap network.

We questioned to what degree AD-associated pathways in the literature exhibit overlap and share genes using enrichment analysis. The 30 most frequently published AD-associated pathways include several significant overlaps. For example, *Alzheimer’s disease* (171 genes) and *Parkinson’s disease* (142 genes) pathways share more than 94 genes (*p*-value < 10E-134). A gene overlap network displays one large component that includes most of the top 30 pathways (Figure 6A). The network contains 199 pairs of pathways with a significant overlap (FDR < 0.05), showing significant gene sharing among the most frequent AD-associated pathways.

In contrast, analysis of KEGG pathways with a set of AD-associated genes from the Open Targets disease gene prioritization system shows enrichment in 8 of the top 30 pathways (FDR < 0.05), including *Parkinson’s disease* and *Huntington’s disease* pathways. We found that pathways that demonstrate enrichment have a higher literature ranking compared with the pathways that are not enriched (*p*-value = 0.0136, Wilcoxon’s rank-sum test; Supplementary Table 8). Surprisingly, the most significantly enriched pathways are not the most highly ranked and include *nicotine addiction*, followed by *neuroactive ligand-receptor interaction*, *cocaine addiction*, *morphine addiction*, *retrograde endocannabinoid signaling*, *GABAergic synapse*, *amphetamine addiction*, *taste transduction*, and *cAMP signaling pathway*. However, after these nine pathways, seven highly ranked pathways also show enrichment in the following order: *Alzheimer’s disease, serotonergic synapse*, *calcium signaling pathway*, *dopAMinergic synapse*, *alcoholism*, *ALS*, and *glutamatergic synapse*.

Using a genetically defined AD gene list, many more *immune* pathways were enriched, including, in the following order of significance: *asthma*, *hematopoietic cell lineage*, *allograft rejection*, *graft-versus-host disease*, *type I diabetes mellitus*, *antigen processing and presentation*, *leishmaniasis*, *intestinal immune network for IgA production*, *autoimmune thyroid disease*, *viral myocarditis*, and *inflammatory bowel disease* (Supplementary Table 9).

### Pathway Co-expression

We examined pathway co-expression to compare the organization of the top 30 ranked KEGG pathways with those that show gene overlap in a network (Figure 6B). The network contained 89 pairs of pathways with a significant correlation (FDR < 0.05). This network contains several links that are not captured in the enrichment-overlap network (Figure 6A). For example, we found that the *proteasome* pathway is co-expressed with both the *AD* and *PD* pathways, yet it has no significant gene overlap.

## Discussion

We have investigated the pathway-specific literature surrounding dementia with a focus on AD by text-mining abstracts with a number of curated dictionaries. The majority of pathways can be associated with AD. Counting only five studies as sufficient support, a total of 91% of KEGG pathways could be related to AD. This number decreases to 88% (10 studies), 63% (50 studies), and still 49% with greater than 100 studies (Supplementary Table 4). Nevertheless, even these low-association pathways have abstracts, which state a manually verified association, for example, the *ferroptosis* pathway only has 13 studies to link it to AD, yet 10 of these score highly as a true association (Supplementary Table 4). On the other end of the pathway rankings, the *PD* pathway has the second highest number of publications after *AD* (Figure 2), due to both diseases often being mentioned together as related even if the study is analyzing only one disease. If we only include studies with an adequate AD word association score, the top ranked pathways after *AD* are *immune system*, *metabolic pathways*, *cholinergic synapse*, *long-term depression* and then *apoptosis*, while *Parkinson’s disease* still ranks high at the fifteenth place (Supplementary Table 4). Alzheimer’s disease is not the only disease in which a large number of pathways have been implicated, but cancer has also been ubiquitously associated across genes and pathways (de Magalhães, 2021).

Pathways ranked by AD-specific studies show a significantly similar order to the ranks from all dementia studies. Using dictionaries, each study was associated with one of the four categories to assess the specificity of pathways to AD, namely, AD, dementia, related dementia, or unrelated dementia. The overall pathway rank from all dementia studies analyzed was highly correlated with the ranks from AD-specific studies (Spearman’s correlation coefficient: 0.98). However, a selection of pathways was overrepresented in one of the four categories. *VEGF signaling* had a high number of studies associated with *related dementias*, as this protein has been associated with both ALS and PD (Xiong et al., 2011; Gao et al., 2014). Excluding pathways with less than 50 total studies, the pathways with the highest proportion (> 88%) of AD-specific studies vs. dementia were *ABC transporters*, *Alzheimer’s disease*, the three *O-glycan*, and two *glycosaminoglycan* pathways (Figure 2). Mutations in the ABC transporter genes have been associated with AD and appear to regulate apoE levels (Wahrle et al., 2004). Defects in the O-glycosylation of APP have been reported in AD, while glycosaminoglycans have been suggested to play a role in plaque deposition and tau aggregation (Stopschinski et al., 2018). The only other pathway to join these if a lower threshold is used (> 5 papers) is *phosphonate and phosphinate metabolism*. From the top 100 ranked pathways, only two significantly change between the overall rank and AD-specific ranks: *HIV1 infection*, which is predominantly associated with AIDS-related dementia, and *Huntington’s disease*, due to a number of studies tagged as *related dementia*.

To estimate, for example, how representative animal models are of the human condition from a pathway perspective, we categorized each study by the type of experiment performed. Interestingly, animal models prioritize different pathways to human studies. For each associated pathway in our analysis, we analyzed the studies by the type of evidence driving the pathway-disease connection (Figure 3). A handful of diseases were linked to AD *via* genetically assigned studies, including the cardiomyopathies and colorectal cancer. The former is, in part, driven by the observation that some known AD genes like *PSEN1* and *BIN1* have also shown association with cardiomyopathy (Li et al., 2006; Hong et al., 2012). The latter has been published with respect to *APOE* and the enzyme COX-2 (Slattery et al., 2005; Wang and DuBois, 2010). However, a handful of studies on this cancer center on genetic testing or the creation of genetic resources, which represent false-positive links between the disease and AD (Heshka et al., 2008). Two *folate* pathways were also scored as both highly genetic and human, which can be attributable to folate often being measured in studies using *APOE*-stratified patients. Also associated is the *spliceosome*, resulting from abstracts that describe a number of different mutations that affect splicing. It is interesting that the top genetic-associated pathways do not match the commonly enriched functions from GWAS despite mostly containing these genes, suggesting that the literature may be biased in which functions are studied.

*Galactose metabolism* represents the most strongly model and animal-associated pathway (Figure 3). This is almost entirely driven by the D-galactose-induced model of aging in mice. However, of note is the distinct lack of either a genetic or a human signal, suggesting that this pathway may not be heavily involved in the human condition. Questions into the suitability of this model to represent aging have already been raised earlier (Parameshwaran et al., 2010). *Long-term potentiation* also displays a model and animal bias. We suggest that this is due to animals more likely being used to study the effects of AD on a cellular level.

The pathways which display the most compelling human-orientated evidence are a mixture of *infection* and *synthesis* pathways (Figure 3). The *HIV1* and *toxoplasmosis* pathways are strongly driven by studies into the AIDS dementia complex, and to a lesser extent into *hepatitis C* and *tuberculosis*, as these two are also composed of comorbidity case studies and induced amyloidosis, respectively. For the *synthesis* pathways, both thyroid hormone and cortisol have their levels reported in patients with Alzheimer’s disease, rather than in animals. Additionally, individuals with Down’s syndrome have higher rates of thyroid disease (Pierce et al., 2017).

The top AD-associated pathways have been consistently the most published over time. To explore the recent trends in research, we developed a yearly rank for all pathways since 1990 (Supplementary Table 6). Most of the top 30 ranked pathways do not significantly change over the last 30 years (Figure 4E). The exceptions include *apoptosis*, which was not heavily published in AD or dementia until 1994 (Figure 4), only 2 years after it was first questioned to be involved in AD in 1992 (Branconnier et al., 1992). *Autophagy* was not published in connection to AD until 1995 (Cissé and Schipper, 1995) and gained strong interest in 2008 (Figure 4D). *NF-kappa B signaling*, *diabetes*, and *insulin*-linked pathways all saw an increase in attention during this period. In 2008, *signaling pathways regulating pluripotency of stem cells* received interest, which saw a further increase in 2014 (Figure 4A). These represent the use of iPSCs as models for studying AD, which were first described in 2006 (Takahashi and Yamanaka, 2006). However, this pathway had a higher than average number of false-positive studies due to methods for the creation of iPSCs, including the pathway’s key terms.

To assess how representative the currently and previously investigated AD drugs are for the pathways they target, we determined their gene targets and therefore the potentially targeted pathways. AD drugs predominantly target immune-related pathways. Clinical trials in AD aiming to either delay the onset or slow disease progression have currently all failed, except for the controversial aducanumab. We examined 69 drugs with this disease-modifying aim to rank all pathways by those most commonly targeted. We found that along with the *Alzheimer’s* pathway, many of these drugs target *immune*, *infection*, and *cancer* pathways. The *serotonergic synapse* is targeted by both symptomatic and disease-altering indications; however, this is in part because the disease-modifying target APP is within this pathway alongside the symptomatic targets, namely, the serotonin transporter and 5-HT1A receptor. Serotonin is frequently targeted because it modulates cholinergic neurons. Two pathways that are not as targeted by AD indications yet score highly in our literature-based ranking are *apoptosis* and the *proteasome*. Additionally, only 2 of the 206 drug-assigned gene targets are also GWAS hits:, namely, atorvastatin that targets *HMGCR* (Moreno-Grau et al., 2019) and avagacestat that targets *APH1B* (Jansen et al., 2019), both of which were investigated to delay the onset of AD (Supplementary Table 10). Given that a greater diversity of pathways are associated with AD *via* the literature than by the investigated drugs, it may be possible that a wide range of aberrant processes are not targeted successfully in clinical trials. However, the list of drugs examined is not an exhaustive list of all clinical trials, many of which lack adequate information including drug name and aim of the drug. Another caveat of examining drugs *via* their targeted pathways is that the bioavailability of the drug or the critical treatment window is not assessed, which are both potential reasons for drug trials being unsuccessful. Additionally, there may be other unknown genes, which are altered by these drugs. We theorize that AD treatments should more comprehensively target associated underlying mechanisms to increase the chances of success.

The top AD-associated pathways are functionally related. We examined the 30 most highly studied KEGG pathways in terms of their gene overlap and co-expression to determine the degree to which pathways that are functionally interrelated are being mentioned for their roles in AD. The neurodegenerative disease pathways, namely, *Huntington’s disease*, *Parkinson’s disease*, and *Alzheimer’s* disease all have genes in common and are co-expressed (Figure 6A). Even when gene overlap is controlled for, they still co-express, suggesting they are functionally interconnected. In contrast, *Parkinson’s* and *Huntington’s disease* pathways are not enriched in the predetermined set of known AD-associated genes. We observed that several pathways are clearly functionally related while not being significantly enriched within each other. These results suggest functional interconnectivity of AD-associated pathways beyond gene-sharing mechanisms and warrant further exploration. For example, the neurodegenerative disease pathways are highly co-expressed with the *proteasome* pathway, which describes a protein-degradation complex, potentially linking in protein misfolding with these disorders. Even when gene overlap is controlled for, they still co-express, suggesting they are functionally interconnected. Each of these is also highly co-expressed with the *proteasome* pathway, which describes a protein-degradation complex, potentially linking protein misfolding with these disorders. In contrast, *Parkinson’s* and *Huntington’s disease* pathways are not enriched in the predetermined set of known AD-associated genes.

Lists of known AD-associated genes do not enrich in the same pathways as the most commonly studied pathways. None of the 10 most significantly enriched pathways with genetically defined AD genes (by GWAS or monogenic AD) are in the top 50 literature-ranked pathways; instead, they represent a number of immune KEGG pathways as well as *type I diabetes mellitus*. There is emerging evidence that AD, indeed, has a strong immune system component (Li et al., 2021), but other aspects of the disease were not enriched in this gene list. The KEGG *Alzheimer’s disease* pathway, which was updated in October 2020 (Kanehisa et al., 2021), contains a small number of the early genetic loci associated with AD and was developed to include processes that are impacted in AD. The *Alzheimer’s disease* pathway is sixteenth of all the significantly enriched pathways for genetically defined AD genes (Supplementary Tables 8, 9). Of the AD genes obtained from Open Targets, the highly ranked pathways *Alzheimer’s disease* and the *GABAergic synapse* also ranked in the top 10 most significantly enriched from this gene list. These two tests suggest that the AD pathway literature is much broader than the pathways highlighted by AD genes.

Regarding design, there are a number of limitations inherent in our study. First and foremost, we used text-mining, which is well known to result in false positives. We attempted to overcome this potential problem by use of thorough manual checking of all pathway associations using three scoring methods (i.e., a pathway score, an AD specificity percentage, and an AD-pathway score) that assessed the pathway dictionaries, the relevance to AD, and the strength of the pathway’s association to AD, respectively (Supplementary Table 4). Second, as negative findings are included in the AMiner dataset, we attempted to filter these with the use of key phrases as part of the dictionary of negative findings. Third, we did not assess the validity of the hypothesis being tested in each study, as this was beyond our available resources. However, we note that the impact factor of the journal, which may serve as a proxy of this validity checking, did not significantly alter the results. Fourth, we only used the third level of KEGG as our core resource to maximize efficiency. As we were mainly focusing on pathway names to represent a range of biological pathways, and many of these overlap with other pathway databases, we believe this approach to be sufficient. Additionally, many pathway enrichment tests use this level. KEGG has been criticized by its lack of inclusion in immune system related pathways. We addressed this limitation by manually adding three immune pathways, namely, *adaptive immune system*, *innate immune system*, and *cytokine signaling in immune system*. The KEGG pathway dictionaries used in this study overlap in similarity to 33% of Gene Ontology (GO) Biological Processes; therefore, a large proportion of GO terms were not tested, as it was outside the scope of this project. Fifth, for the drug and network analysis, we relied on the genes assigned to each pathway by KEGG or Reactome, which was not the case for the main analysis, so some caution must be used when comparing these results. Sixth, our analysis required the abstract of the publication to be available, which excluded a number of studies, either because they lacked an accessible abstract or because the pathways associated with AD were not explicitly stated in the abstract. Pathways like *Fc epsilon RI signaling* score poorly in our project, yet a google scholar search of this with *Alzheimer’s disease* finds a number of additional studies. This limitation could have been addressed by including more of the study beyond the abstract in the text-mining, but this would have greatly increased the number of false-positive associations, particularly for pathways like *biotin metabolism*, as biotin is commonly used in laboratory experiments.

## Conclusion

In this study, we have assessed the biological pathways that are maximum reported in AD using text-mining of > 200,000 publications. We find that a total of 91% of all KEGG pathways could be connected to AD *via* the literature, suggesting that – if our results are correct – not only that this disorder is incredibly complex, but that its functional reach is also nearly global. For the last 30 years, the same set of core pathways have been consistently related to AD, suggesting that the main focus of AD research has not radically altered over time despite great technological advances made in the same time period. Remarkably in this context, known AD gene enrichment is not reflected in terms of the most frequently studied pathways. We hope that researchers will use these results to assess their own prioritized pathways.

## Data Availability Statement

The datasets analyzed during the current study are available in the AMiner 616 repository, at www.aminer.org/open-academic-graph. The main results can be explored at www.adpathways.org while the files can be downloaded from https://dataverse.harvard.edu/dataverse/PathwaysAD. The code used for performing enrichment analysis and network analysis can be found at https://github.com/pouryany/Mining_AD_Pathways.

## Author Contributions

SM and PN analyzed the data. KK provided the drug synonyms database. SM, PN, YP-J, IV, and WH contributed to method design. DP and RT provided data and disease expertise. SM, PN, LB, and WH contributed to writing the manuscript. All authors read and approved the final manuscript.

## Conflict of Interest

The authors declare that the research was conducted in the absence of any commercial or financial relationships that could be construed as a potential conflict of interest.

## Publisher’s Note

All claims expressed in this article are solely those of the authors and do not necessarily represent those of their affiliated organizations, or those of the publisher, the editors and the reviewers. Any product that may be evaluated in this article, or claim that may be made by its manufacturer, is not guaranteed or endorsed by the publisher.
