# Supplementary material for: Most Pathways Can Be Related to the Pathogenesis of Alzheimer’s Disease
Source: Front Aging Neurosci. 2022 Jun 24;14:846902. doi: 10.3389/fnagi.2022.846902 (PMC9263183; doi:10.3389/fnagi.2022.846902)
Supplement: Supplementary file 1 [file Data_Sheet_1.PDF]

Supplementary Material

1 Supplementary Methods

1.1 Filtering the AMiner dataset

AMiner does not contain preprints, for example, from bioRxiv, but does contain some conference abstracts that associate with a journal (Wan et al., 2019). These were removed from the analysis by matching the *conference*, *symposium,* or *poster* in the abstract title. We kept only papers with an abstract (kept n=40,743,723), containing the terms Alzheimer or dementia (kept n=206,324). Next we filtered for quality measures; duplicate papers were removed as determined by the Microsoft Academic Graph tool for assessing similarity designed for this exact dataset (Wang et al., 2019). The graph tool enabled us to exclude papers with a higher than 90% similarity in their title, authors, journal, date and abstract (removed n=8,110). We also identified duplicate papers by identifying between the first 200 and last 200 characters of the abstract (removed n=1,681) and those with the same DOI under the condition that this ID had greater than 7 characters (removed n=3,681). Papers with abstracts under 63 characters were excluded (removed n=432), as well as poster sessions (removed n=52) and abstracts describing Alzheimer's or dementia as an exclusion criterion for their study (removed n=188). Lastly, if the number of keywords were greater than 30, then all keywords were removed from the paper entry.

1.2 Pathway dictionaries

Each pathway was individually curated by a biologist (SLM) to ensure that all related search terms and phrases were included in the dictionary of searches, assisted by lemmatization (to form roots of terms). Within the AMiner dataset, we extracted semantically similar words that commonly occurred with the pathway terms, and in contrast, those which altered the meaning of the pathway term to something irrelevant. These irrelevant terms also included a dictionary specific to negative findings. We queried the filtered AMiner database with these terms to estimate the total number of papers associated with each pathway.

For pathways like *oxidative phosphorylation*, an exact match of the whole term was required, whereas, *glycolysis/gluconeogenesis* allowed for fuzzy matching. We recorded how many abstracts matched the full terms verses any fuzzy matching or a significant partial match for each pathway (Supplementary Table 4). Two-part pathways, for example *pantothenate and CoA biosynthesis*, are more likely to have partial association: where either *pantothenate synthesis* or *CoA synthesis* might be present, but rarely both. Multipart pathways might have skewed hits, for example, *fructose and mannose metabolism* has the majority of its associations from *fructose*.

1.3 Handling of similar named pathways

For pathways that are very similar to each other, for example, *fatty acid metabolism* and *fatty acid biosynthesis*, it was decided that the aim of the project was to recapitulate the real-world scenario of a researcher investigating a prioritized pathway. Given that a researcher would be interested in all aspects of fatty acid processes, even if they only prioritized one, this study allowed these terms to heavily overlap. For example, papers on fatty acid contributed to the score of both *fatty acid*

metabolism and *fatty acid biosynthesis*. However, to assess if there were more papers that specify *metabolism* over *biosynthesis*, we removed papers that were specific to *metabolism* from the *fatty acid biosynthesis* score, and vice versa. This explains why the scores are very similar, but not identical. None of the specific fatty acid pathways are associated significantly more than the other.

1.4 Pathway score

The pathway score was based on 10 or more abstracts which were manually read to decide yes/no if the abstract's pathway mention was true or not. If more than 10% of papers failed to match the appropriate pathway, the dictionaries were altered until an adequate score was achieved with 10 new random abstracts (≤10%). A score of *high* describes a pathway where all 10+ abstracts mention that pathway adequately. A *good* score describes a pathway with false positives in the range of 1-9% whereas an *ok* ranking is a 10% false positive rate. Improving the dictionaries mostly consisted of adding terms or key phrases to the removal list e.g., abstracts are removed if they contain the phrase *no association was found*, and similar terms.

1.5 Evidence dictionaries

To categorize the type of evidence driving an association, we used dictionaries to represent *genetic*, *model*, *human* and *animal* studies. Some abstracts were categorized into more than one of these groups. Dictionaries were creating using topic modelling using latent Dirichlet allocation (Blei, 2012). Words that were duplicated, for example, *brain* in both *animal* and *human* categories were removed, as were terms that were manually assessed to be not relevant to the category, for example, *learning* was removed from the *animal* dictionary. Abstracts with 5 or more dictionary terms (from any category) had to have more than 60% of words from a single category to be labelled in that group. For 2-4 category terms, the same cutoff applied but abstracts were additionally labelled as *low* to mark them as low certainty. Additionally, at least one of the words had to be a strong indication word, for example, *rat* or *mouse* for the *animal* category.

1.6 Evidence driving Alzheimer's disease association

Most human subject-focused journals had 80-99% of their papers containing human-associated research, except for Human Molecular Genetics which totaled only 59%. Examining further we found that the 6th most common word (discounting prepositions) was *human* while the 7th and 15th was *mice* and *mouse* respectively. Unlike the other human-related journals we tested, this journal contained a number of animal studies. For the animal-orientated journals, our dictionaries classified more papers as *animal* over *human*, however, this was less pronounced than the *vice versa* in the human journals (Supplementary Figure 1).

Whilst the animal/human and genetic/model dictionaries performed adequately for our study, the *in vivo* vs *in vitro* comparison did not (Supplementary Figure 1). We could not define a set of terms and phrases that differentiated these two groups. Roughly half of all papers from the three selected journals fell under the *both* grouping. Only 9% of papers from the journal In Vivo were labelled as *in vivo*. Consequently, we decided to drop this comparison from the study.

1.7 Number of pathway associations per abstract

Two of the pathways which we labeled as not associated with AD, included *antifolate resistance* and *maturity onset diabetes of the young*. These both had a single paper identified, where either the

pathway or Alzheimer's disease (AD) were only mentioned in the keywords and not in the abstract, therefore the connection could not be established. To examine the number of pathways associated with each paper, we plotted a histogram (Supplementary Figure 2). This excluded the *Alzheimer's disease* pathway.

1.8 Dementia dictionaries

There were four categories of dementia stratification dictionary: AD, related dementia, unrelated dementia and dementia. To be categorized as *AD*, an abstract had to include *Alzheimer* and could additionally include *APP*, *tau*, *APOE*, *hippocamp*, *memory*, *MCI* and 6 different ways of writing *amyloid*. It could not include more than 2 words from the related dictionary: *FTD*, *frontotemporal*, *vascular*, *Lewy*, *Huntington*, *HD*, *Parkinson*, *PD* or more than 1 word from the unrelated dictionary: *HIV*, *AIDS*, *Rett*, *Pick*, *neuropathy*, *dystrophy*, *spongiform*, *Wallerian*, *retinal degeneration*, *macular degeneration*, *prion disease*, *corticobasal degeneration*, *multiple sclerosis*, *pressure hydrocephalus*, *posterior cortical atrophy*, *palsy*, *Creutzfeldt*, and *Fahr*. If multiple diseases were discussed in an abstract, then it was categorized under the *dementia* label.

1.9 Testing the dementia dictionaries

To understand biases in patient subtype stratification, we created dictionaries for each category: Alzheimer's disease, dementia, related dementia, and unrelated dementia. These dictionaries were tested on journals that represent each category (Supplementary Figure 3). For journals specific to AD, over 70% of papers are categorized as AD papers (Supplementary Figure 3A), while journals from related disorders like Parkinson's disease are labelled as 'related dementia' in 70-90% of papers (Supplementary Figure 3C). If a journal has a non-specific disease interest like Translational Neurodegeneration or Dementia, the papers are assigned to all categories at varying amounts (Supplementary Figure 3B and D). Lastly, we included journals that might have an unrelated dementia or no relation to this study, for example, Journal of Rare and Uncommon Diseases, which except for the journal Prion, all scored very low on AD, related dementia or dementia categories (Supplementary Figure 3E).

1.10 Drug curation

A number of the drugs trials undertaken in AD do not report whether the treatment is theorized to be symptomatic or disease-modifying. We used Alzforum therapeutics (ALZFORUM, 2021) to manually curate the drugs into either of these two categories, or sometimes into both (Supplementary Table 10). This categorization was not always clear from the literature and, in fact, some drugs were initially investigated as disease-modifying but then changed to symptomatic, highlighting the lack of understanding in the cause of AD.

2 Supplementary Tables and Figures

2.1 Supplementary Tables

Supplementary Table 1. Scoring AD-pathway connections. When assessing a selection of 2-20 papers per pathway for their association to AD, we developed a scoring system to judge the strength of the evidence (Supplementary Table 1). Direct and obvious pathway associations scored 1 while loose, indirect connections scored 5. The type of dementia mentioned was also included in this score. AD papers could score the highest while related dementia, like frontotemporal dementia, could score a maximum of 3 and unrelated dementias could only achieve a 4.

Supplementary Table 2. AD-associated genes as determined by Open Targets. Using the Open Targets Platform (Carvalho-Silva et al., 2019; Open Targets, 2021) we searched for Alzheimer's disease and selected for genetic associations, drugs, pathways & systems biology and RNA expression. We downloaded the available genes and filtered for those with a greater than 0.1 overall score. These were used to perform gene enrichment tests. All the scores within this table are directly from the Open Targets website. We also filtered genes for those within the KEGG database, leaving 308 genes.

Supplementary Table 3. Genetically-associated AD genes from GWA and familial studies. Curated list of genes published with Alzheimer's disease improved from previous work(Pita-Juárez et al., 2018)**.**

Supplementary Table 4. Total paper counts and scores for each pathway's association with dementia and AD via the literature. We investigated KEGG pathways plus three Reactome pathways and three added terms: *immune system*, *cancer* and *diabetes*. There was no significant difference between the pathway ranks from the whole of the dementia literature compared to AD-specific papers. The pathway_score was based on ~10 papers judging how often the paper fully related to the pathway. The AD-pathway_score is the average score for papers where the pathway's association to AD was judged based on 2-10 papers. More papers were assessed for pathways with fewer total papers. The AD_word_score is the average number of AD-specific words used in each paper assigned to each pathway, based on all associated papers.

Supplementary Table 5. Evidence breakdown for each pathway for genetic vs model and animal vs human. We excluded pathways with less than 100 papers from our analysis. There are overlaps between these comparisons, for example papers with a human tag could also have a genetics tag.

Supplementary Table 6. Pathway ranks annually since 1990. We ranked pathways based on their yearly publication record in association with dementia. If two+ pathways had the same number of papers, then the ranks were averaged to keep all years on the same scale.

Supplementary Table 7. The pathways most targeted by symptomatic and disease-modifying treatments. We used Open Targets (Open Targets, 2021) and Therapeutic Target Database (Wang et al., 2020) to assign gene targets to drugs. Drugs were only counted once per pathway even if they targeted it via multiple genes. We did not correct for pathway size as larger pathways do not require more genes to be targeted to cause an effect.

Supplementary Table 8. Enrichment for AD genes from Open Targets. Supplementary Table 2 was used as the input gene lists for this enrichment test. Only KEGG was used in this analysis.

Supplementary Table 9. Enrichment for AD GWAS genes. Supplementary Table 3 was used as the input gene lists for this enrichment test. Only KEGG was used in this analysis.

Supplementary Table 10. Curated drugs from clinicaltrials.gov. Categories were symptomatic, delay onset, slow progression, memory enhancing or a combination of any two.

2.2 Supplementary Figures

Supplementary Figure 1. Performance of our evidence dictionaries on target journals. Journals specific to one of the 6 evidence types (human, animal, model, genetic, in vivo, in vitro) were selected to examine our evidence dictionaries. Bars are aligned along the midpoint of the neutral 'both' category so that a direct comparison can be made of the two competing groups. The missing category from this chart is for papers that contained none of the key dictionary terms, but this value can be inferred by subtracting the total bar quantity from 100. (A) We compared animal with human studies. Human-orientated journals show a stronger preference for using words from our human-defined dictionary compared to animal journals with the terms from our animal dictionaries. In part, this is because many human diseases are modelled in animals which causes the human aspects of the disease to be mentioned in abstracts. Additionally, the animals included in our dictionaries were animals used commonly in AD research, for example, rodents, zebrafish and monkeys. However, the animal literature, particularly the journal Animal Genetics, covers many other species, specifically research on cattle. Similarly, when comparing (B) genetic vs model, this journal was predicted as containing many more genetic papers than model for the same reason. Likewise, for Human Molecular Genetics there are mostly genetic papers (65%) yet still a number of model papers (21%). We checked a subset of these and confirmed this to be the correct assignment. Of note is that while human models (e.g., cells) are included in the dictionaries under the model umbrella, studies directly on patients do not, which is exemplified by the results for Human Molecular Genetics. Both human and animal studies were included under the 'model' category. (C) in vitro and in vivo studies. We could not adequately differentiate between these two categories with most papers assigned to the both label.

Supplementary Figure 2. (A) Histogram of the number of pathways associated with every paper after removing the Alzheimer's disease pathway. 60% of papers had 1 or more pathways associated with it. (B) The same figure with a maximum frequency of 30. Less than 1% of papers had a high number of pathways (>15) assigned. Looking at these in detail, many of them have several pathway terms as keywords as well as listing a number of processes in the abstract that are associated with Alzheimer's.

Supplementary Figure 3. Percentage of papers from different journals that are categorized by the dementia dictionaries on journals specific to each category. (A) Alzheimer's journals. (B) Alzheimer's and other dementia journals. (C) Related dementia journals like Parkinson's disease. (D) Neurodegeneration journals. (E) Unrelated dementia or rare disease journals.

3 References

ALZFORUM (2021). Therapeutics | ALZFORUM. Available at: https://www.alzforum.org/therapeutics [Accessed May 28, 2021].

Blei, D. M. (2012). Probabilistic topic models. *Commun. ACM* 55, 77–84. doi:10.1145/2133806.2133826.

Carvalho-Silva, D., Pierleoni, A., Pignatelli, M., Ong, C., Fumis, L., Karamanis, N., et al. (2019). Open Targets Platform: new developments and updates two years on. *Nucleic Acids Res* 47, D1056–D1065. doi:10.1093/nar/gky1133.

Open Targets (2021). Open Targets Platform. Available at: https://platform.opentargets.org/ [Accessed March 13, 2021].

Pita-Juárez, Y., Altschuler, G., Kariotis, S., Wei, W., Koler, K., Green, C., et al. (2018). The Pathway Coexpression Network: Revealing pathway relationships. *PLoS Comput Biol* 14, e1006042. doi:10.1371/journal.pcbi.1006042.

Wan, H., Zhang, Y., Zhang, J., and Tang, J. (2019). AMiner: Search and Mining of Academic Social Networks. *Data Intellegence* 1, 58–76. doi:10.1162/dint_a_00006.

Wang, K., Shen, Z., Huang, C., Wu, C.-H., Eide, D., Dong, Y., et al. (2019). A Review of Microsoft Academic Services for Science of Science Studies. *Front. Big Data* 2, 45. doi:10.3389/fdata.2019.00045.

Wang, Y., Zhang, S., Li, F., Zhou, Y., Zhang, Y., Wang, Z., et al. (2020). Therapeutic target database 2020: enriched resource for facilitating research and early development of targeted therapeutics. *Nucleic Acids Res* 48, D1031–D1041. doi:10.1093/nar/gkz981.
