# Supplementary figures and images for: Most Pathways Can Be Related to the Pathogenesis of Alzheimer’s Disease
Source: Front Aging Neurosci. 2022 Jun 24;14:846902. doi: 10.3389/fnagi.2022.846902 (PMC9263183; doi:10.3389/fnagi.2022.846902)

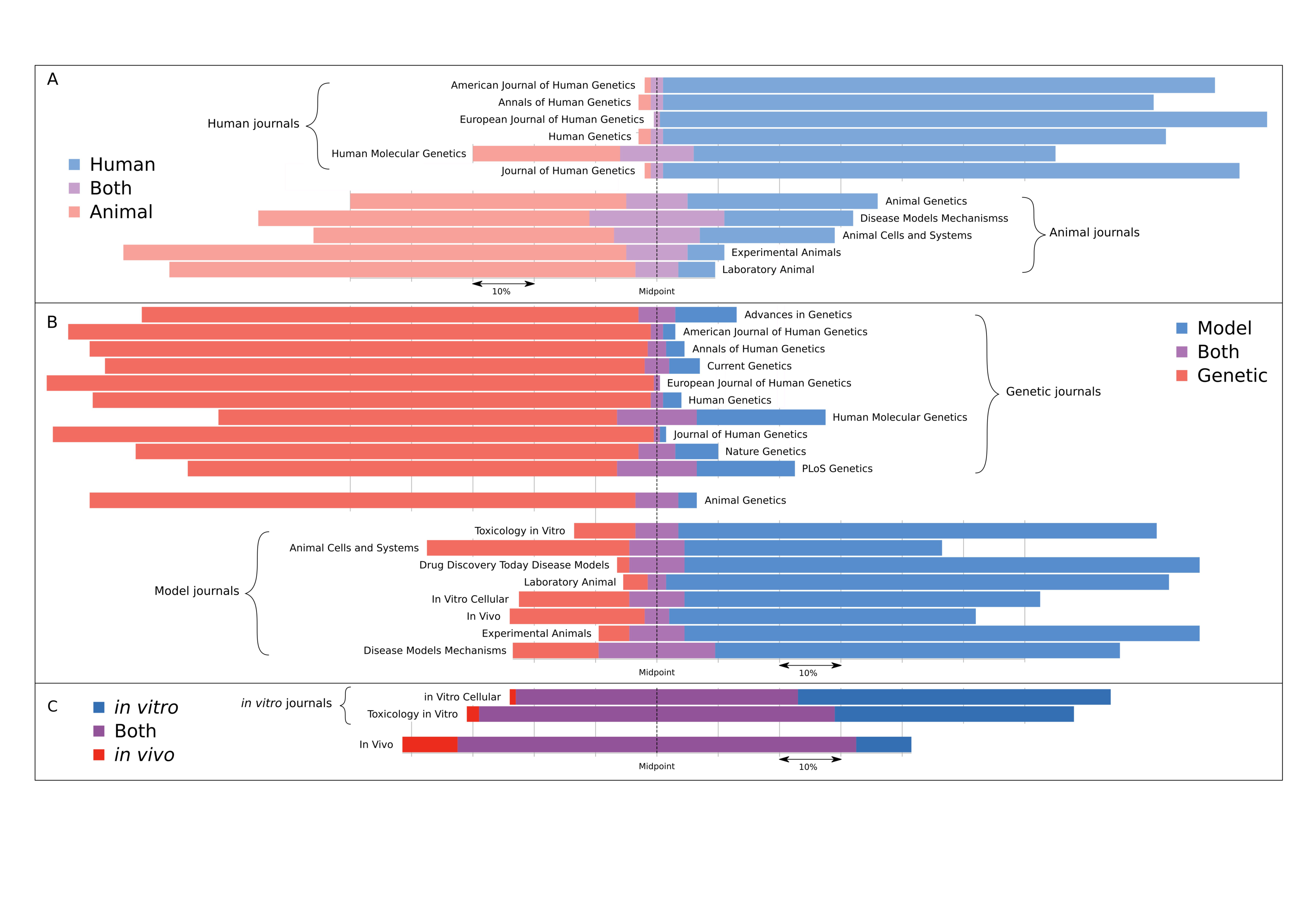

Supplement: Supplementary file 3 [file Image_1.TIFF]

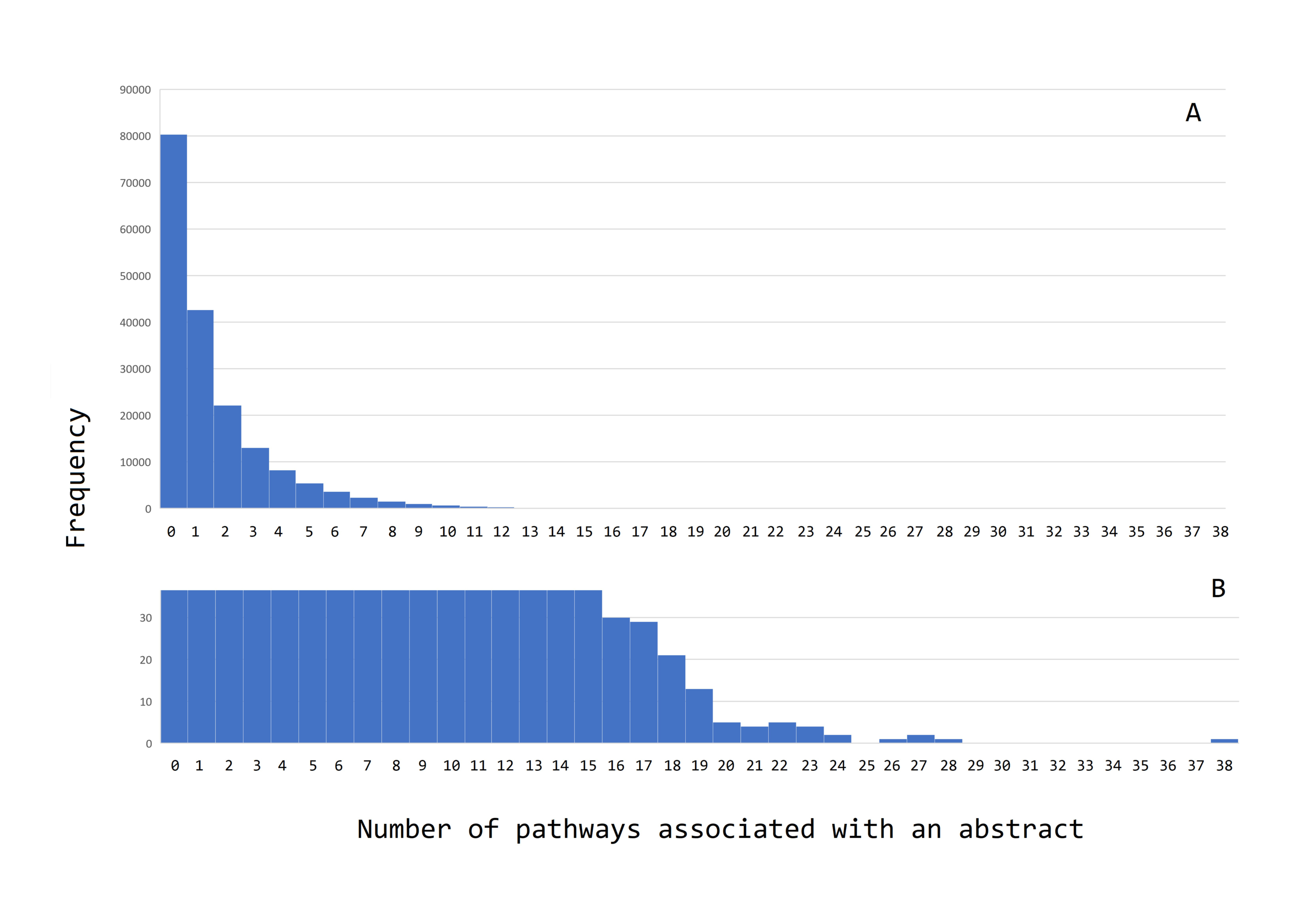

Supplement: Supplementary file 4 [file Image_2.TIFF]

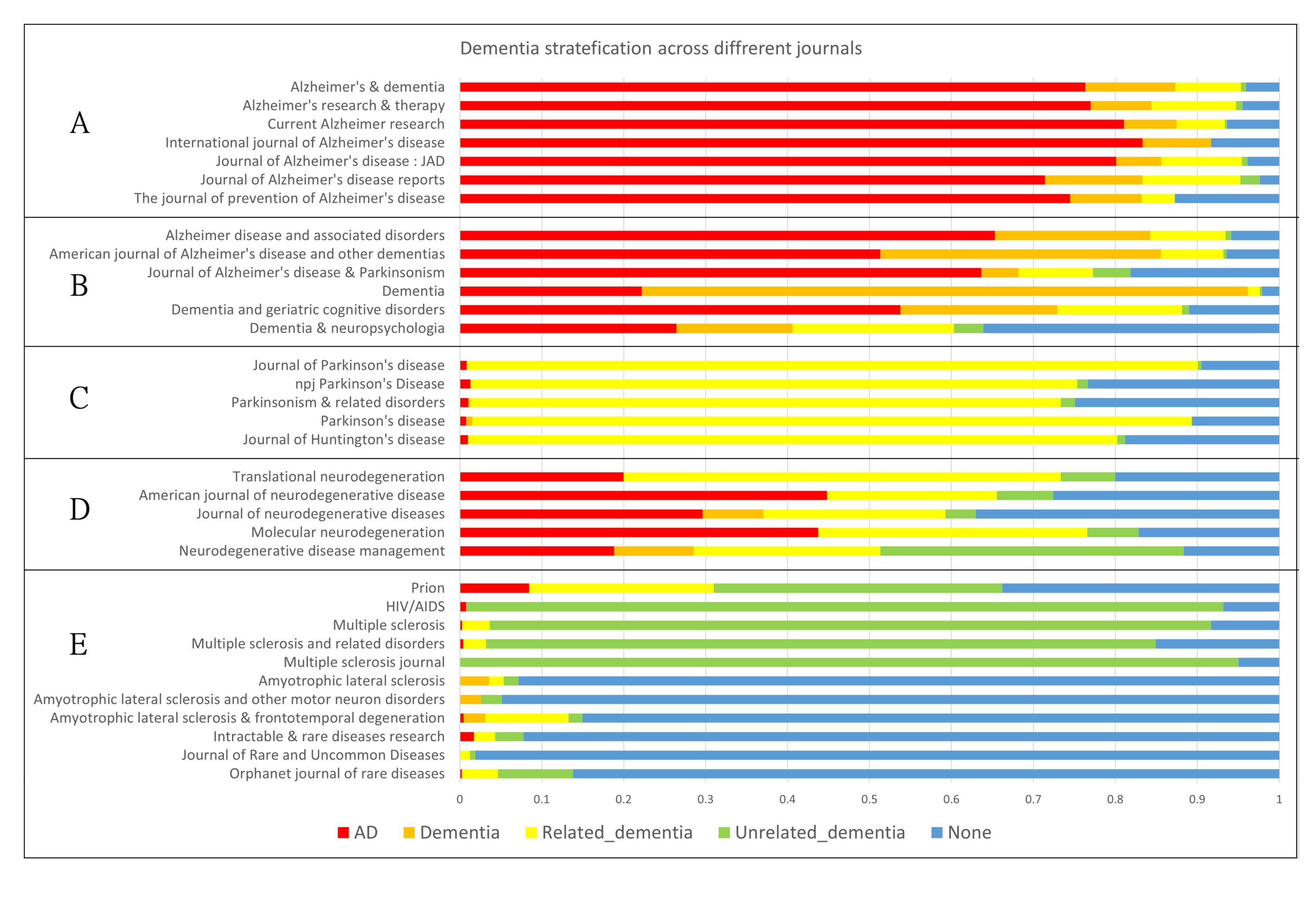

Supplement: Supplementary file 5 [file Image_3.TIFF]
